# Plasmonic Quantum Nanocomposite-Modified Multimode–Multicore Fiber for Sensitive Optical Biosensing of Triclosan

**DOI:** 10.3390/nano16211339

**Published:** 2026-10-22

**Authors:** Jiaqi Gao, Ben Niu, Mengmeng Zhang, Feilong Gao, Yiyan Xie, Ragini Singh, Rui Min, Xian Zhou, Carlos Marques, Bingyuan Zhang, Santosh Kumar

**Affiliations:** 1 Shandong Key Laboratory of Optical Communication Science and Technology, School of Physics Science and Information Technology, Liaocheng University, Liaocheng 252059, China; 2 Postdoctoral Research Station, Vital Optics Technology Co., Ltd., Chuzhou 239064, China; 3 Vital Materials Co., Ltd., Beijing 100192, China; 4 Liaocheng Science and Technology Information Research Center, No. 16 Huanghe Road, High-Tech District, Liaocheng 252001, China; 5 Department of Biotechnology, Koneru Lakshmaiah Education Foundation, Vaddeswaram 522302, Andhra Pradesh, India; 6 Bay Area School of Applied Psychological Sciences, Beijing Normal University, Zhuhai 519087, China; 7 Faculty of Psychology, Beijing Normal University, Beijing 100875, China; 8 School of Computer & Communication Engineering, University of Science & Technology Beijing, No. 30, Xue Yuan Road, Haidian District, Beijing 100083, China; 9 CICECO–Aveiro Institute of Materials & Physics Department, University of Aveiro, Campus Universitário de Santiago, 3810-193 Aveiro, Portugal; 10 Department of Physics, VSB–Technical University of Ostrava, 70800 Ostrava, Czech Republic; 11 Centre of Excellence for Nanotechnology, Department of Electronics and Communication Engineering, Koneru Lakshmaiah Education Foundation, Vaddeswaram 522302, Andhra Pradesh, India; santosh@kluniversity.in

**Keywords:** triclosan detection, plasmonic quantum nanocomposites, multimode–multicore fiber biosensor, environmental pollutant monitoring, life below water, clean water and sanitation

## Abstract

The incorporation of hybrid nanomaterials and fiber architectures into fiber-optic biosensors has proven to be effective in improving sensing performance. This study developed a plasmonic biosensor using a tapered multimode fiber (MMF)–multicore fiber (MCF)–MMF structure functionalized with a nanocomposite of gold nanoparticles (AuNPs), tungsten disulfide quantum dots (WS_2_-QDs), and multi-walled carbon nanotubes (MWCNTs) for selective triclosan detection. Combining WS_2_-QDs and MWCNTs with AuNPs leverages their individual advantages, creating an active sensing interface with abundant analyte-binding sites, thereby substantially improving responsiveness. The composite-modified structure also promotes higher-order mode generation, which is critical for sensitivity enhancement. When optically excited, the periodic plasmonic geometry produces strong evanescent fields that intensify interactions between the immobilized nanocomposite and target molecules. Transmission spectroscopy demonstrated that the biosensor achieved a detection limit of 3.87 µM within the 0–40 µM range. These results show that the strategic combination of WS_2_-QDs and MWCNTs with AuNPs significantly amplifies localized surface plasmon resonance (LSPR) effects. This architecture offers a promising approach for developing sensitive fiber-optic biosensors, particularly for environmental monitoring of agents like triclosan.

## 1. Introduction

Triclosan is a widely used synthetic antimicrobial compound found in various personal care and hygiene products, including toothpaste, hand sanitizer, body wash, and soap. It appears as a white crystalline powder with a slight aromatic odor, is slightly soluble in water, and readily soluble in organic solvents such as ethanol and acetone. Triclosan exhibits high chemical stability without decomposition below 280–290 °C, remains stable under acidic and alkaline conditions, and has a density of approximately 1.4214 g/cm^3^ [1]. Triclosan inhibits InhA, an essential enoyl-acyl carrier protein reductase, thereby interfering with mycolic acid biosynthesis and disrupting cell-wall formation in Mycobacterium tuberculosis [2]. Since the outbreak of the coronavirus in 2019, the global demand for disinfectants has increased significantly, and the global production of triclosan has grown linearly [3]. Regulatory standards set by the European Commission, Canada, the United States, and China stipulate that the maximum allowable concentration of triclosan in soaps, deodorants, and oral care products must not exceed 0.3% (*w*/*w*), and Japan has further reduced the allowable limit in cosmetics to 0.1% [4]. Considering the molecular weight of triclosan (289.55 g/mol), 0.3% and 0.1% correspond approximately to 10.4 and 3.45 mM, respectively, assuming a density close to 1 g/mL. These concentrations are substantially higher than the 0–40 μM working range investigated in this study; therefore, the experimental range represents a diluted analytical concentration range rather than the concentration of undiluted commercial formulations. Excessive triclosan concentrations can interfere with the human endocrine system (such as causing thyroid dysfunction), affect reproductive development, and may also increase the risk of antibiotic resistance, posing potential harm to human health. To mitigate these health risks, reliable detection of triclosan is imperative; given its widespread presence in consumer products, it is necessary to monitor its concentration in these products.

Currently, the detection of triclosan mainly relies on instrumental analysis methods such as high-performance liquid chromatography (HPLC) [5,6], gas chromatography–mass spectrometry (GC-MS) [7,8], and liquid chromatography–tandem mass spectrometry (LC-MS/MS) [9,10]. Although these methods are accurate and effective, they also face some limitations, such as high costs, complex instruments, lengthy procedures, specialized operator training, and large sample volume requirements. In recent years, optical fiber biosensors have rapidly gained popularity due to their advantages, such as low cost, small size, light weight, resistance to electromagnetic interference, superior sensing performance, and reliable operation in harsh environments. However, the application of fiber-optic biosensors in triclosan detection remains unexplored, so further research is needed to assess their potential in this field.

The rapid development of nanotechnology has greatly promoted the application of fiber-optic biosensors based on precious metal nanoparticles (MNPs) [11], especially through their localized surface plasmon resonance (LSPR) properties. Recent advances have further demonstrated the potential of fiber-optic LSPR biosensors that combine tailored fiber structures with plasmonic nanomaterials for sensitive and label-free detection [12]. These biosensors have demonstrated great potential in various detection fields, mainly due to the unique LSPR sensitivity of precious MNPs to changes in the environmental refractive index (RI) [13]. Recent studies have also highlighted the development of novel fiber architectures and nanomaterial-assisted strategies to further improve the performance of fiber-optic SPR/LSPR biosensors [14]. Their basic principle relies on the evanescent waves (EWs) generated during the propagation of light in optical fibers. When incident light meets the critical angle at the interface between two dielectric media, total internal reflection occurs, creating an exponentially decaying electromagnetic field (EWs) that penetrates into the lower RI medium. This evanescent field interacts with MNPs near the fiber surface, inducing collective oscillations of conductive electrons (surface plasmons) within the nanoparticles (NPs) [15]. The coupling between the EWs and MNPs’ surface plasmons generates intense localized electromagnetic fields, forming the basis for LSPR-based sensing applications. The sensing principle of plasmonic sensors relies on monitoring wavelength shifts in the LSPR absorption peak of MNPs. Specifically, functionalized probes with tailored ligands enable selective binding of target analytes to NP surfaces, which modifies the local RI near the nanostructures. RI changes cause measurable shifts in the LSPR spectral peaks [16,17], thereby converting biomolecular interactions into quantifiable optical signals through analysis.

In addition, the two-dimensional spatial structure and unique physicochemical properties of nanomaterials (NMs) have significantly promoted the development of optical fiber biosensing [18]. Surface functionalization of optical fibers with plasmonic nanomaterials can increase the effective interaction between the evanescent optical field and the surrounding recognition layer, while providing additional surface area and functional groups for immobilizing biorecognition elements. Such interface engineering facilitates the conversion of molecular recognition events into measurable changes in the LSPR response. In the field of LSPR sensing, precious MNPs such as gold (Au) remain the preferred material [19]. Gold nanoparticles (AuNPs) exhibit characteristic light absorption in the visible spectrum when excited by light, while gold nanostructures exhibit superior chemical stability and biocompatibility [20]. The strong affinity of thiol groups for gold surfaces and the facile functionalization of AuNPs with biomolecules make AuNPs useful interfaces for antibody immobilization and plasmonic biosensing [21,22]. For the fixation of triclosan-specific antibodies, different NMs have unique advantages: MWCNTs have excellent electrical conductivity and can promote rapid electron transfer [23]. Transition metal disulfide compounds (TMDs) [24], as a class of two-dimensional materials with structural similarity to graphene [25], have emerged as promising materials for optoelectronic applications. Compared with graphene, these layered semiconductors demonstrate enhanced photon absorption capabilities due to stronger light–matter interactions, which has driven substantial progress in photodetector development. As a representative TMD material, tungsten disulfide (WS_2_) demonstrates exceptional thermal stability across diverse operational conditions [26]. Recent studies show that hybrid nanostructures combining WS_2_ atomic layers with AuNPs can synergistically enhance LSPR excitation efficiency through interfacial coupling effects [27]. In addition to grating- and metasurface-based configurations, the lab-on-fiber paradigm also encompasses multi-tapered plasmonic architectures, which offer the advantages of simplified fabrication and robust LSPR excitation [28].

Recent immunoassay-based studies have also demonstrated the feasibility of antibody-derived molecular recognition for selective triclosan determination in water samples [29]. Despite the progress in triclosan detection, relatively limited attention has been given to integrating selective molecular recognition with a tapered fiber-optic LSPR platform and a multifunctional nanocomposite sensing interface. In particular, the individual contributions of different functional nanomaterials to the optical response of a fiber-based triclosan biosensor have not been sufficiently clarified. To address this gap, we developed a label-free plasmonic biosensor based on a tapered multimode fiber (MMF)–four-core fiber (FCF)–MMF structure functionalized with AuNPs, MWCNTs, and WS_2_-QDs, followed by the immobilization of a triclosan-specific monoclonal antibody. The tapered fiber structure enhances the interaction between the guided optical field and the surrounding sensing interface through the generation of evanescent waves, while AuNPs provide the LSPR transduction mechanism. MWCNTs and WS_2_-QDs were incorporated to construct a multifunctional nanocomposite interface, and their individual contributions were further evaluated experimentally using AuNP-only, AuNP/MWCNT, AuNP/WS_2_-QD, and AuNP/MWCNT/WS_2_-QD configurations. The combination of the tapered fiber architecture, ternary plasmonic nanocomposite, and antibody-based molecular recognition therefore represents the main design feature of this work. The sensing performance was evaluated through calibration, reproducibility, selectivity, regeneration/reuse, and the analysis of commercial toothpaste and facial cleanser samples.

## 2. Experimental Section

### 2.1. Materials and Methods

In this work, triclosan was purchased from Macklin (Shanghai, China). Mouse anti-triclosan monoclonal antibody was purchased from Beijing Baiotai Company (Beijing, China). The experimental setup utilized two types of optical fibers: multi-mode fiber (MMF, 62.5 µm core/125 µm cladding) and four-core fiber (FCF, 8.0 µm core/125 µm cladding), commercially sourced from Shenzhen EB-link Technology Co. (Shenzhen, China) and Fiber Core Ltd. (Southampton, UK), respectively. An AuNPs colloid was synthesized through chemical reduction in HAuCl4 using trisodium citrate as a stabilizing agent in deionized water. MWCNTs were purchased from Nanjing Xianfeng Nanomaterial Technology Company Ltd. (Nanjing, China), and they were dispersed in an N,N-dimethylformamide (DMF) solution. WS_2_-QDs were purchased from Nanjing Xianfeng Nanomaterial Technology Company Ltd., and they were dispersed in deionized water. The product was specified as WS_2_ quantum dots with a size of <10 nm according to the manufacturer’s certificate of analysis. The sensing probe fabrication involved specialized fiber processing equipment, including a Fujikura FSM-100P+ fusion splicer (Fujikura, Tokyo, Japan) and CMS (Jacksonville, FL, USA). Precision cleaving of optical fibers was achieved using a CT106 large-core fiber cutter (Fujikura, Japan). For NP characterization, the optical absorption properties of AuNPs were analyzed through UV-Vis spectroscopy (Hitachi U-3310 spectrophotometer, Tokyo, Japan), while morphological analysis of the nanomaterials (NMs) was conducted via high-resolution transmission electron microscopy (HR-TEM, Thermo Fisher Talos L120C, Waltham, MA, USA). Surface coating integrity of the NMs on the probe was verified through scanning electron microscopy (SEM, Carl Zeiss Gemini, Jena, Germany) using a high-energy electron beam. Optical transmission performance evaluation employed a tungsten–halogen illumination system (Ocean Optics HL-2000, Dunedin, FL, USA) coupled with a USB2000+ spectrometer (Ocean Optics, FL, USA) for spectral acquisition.

### 2.2. Sensing Mechanism of the Probe

**Figure 1:**
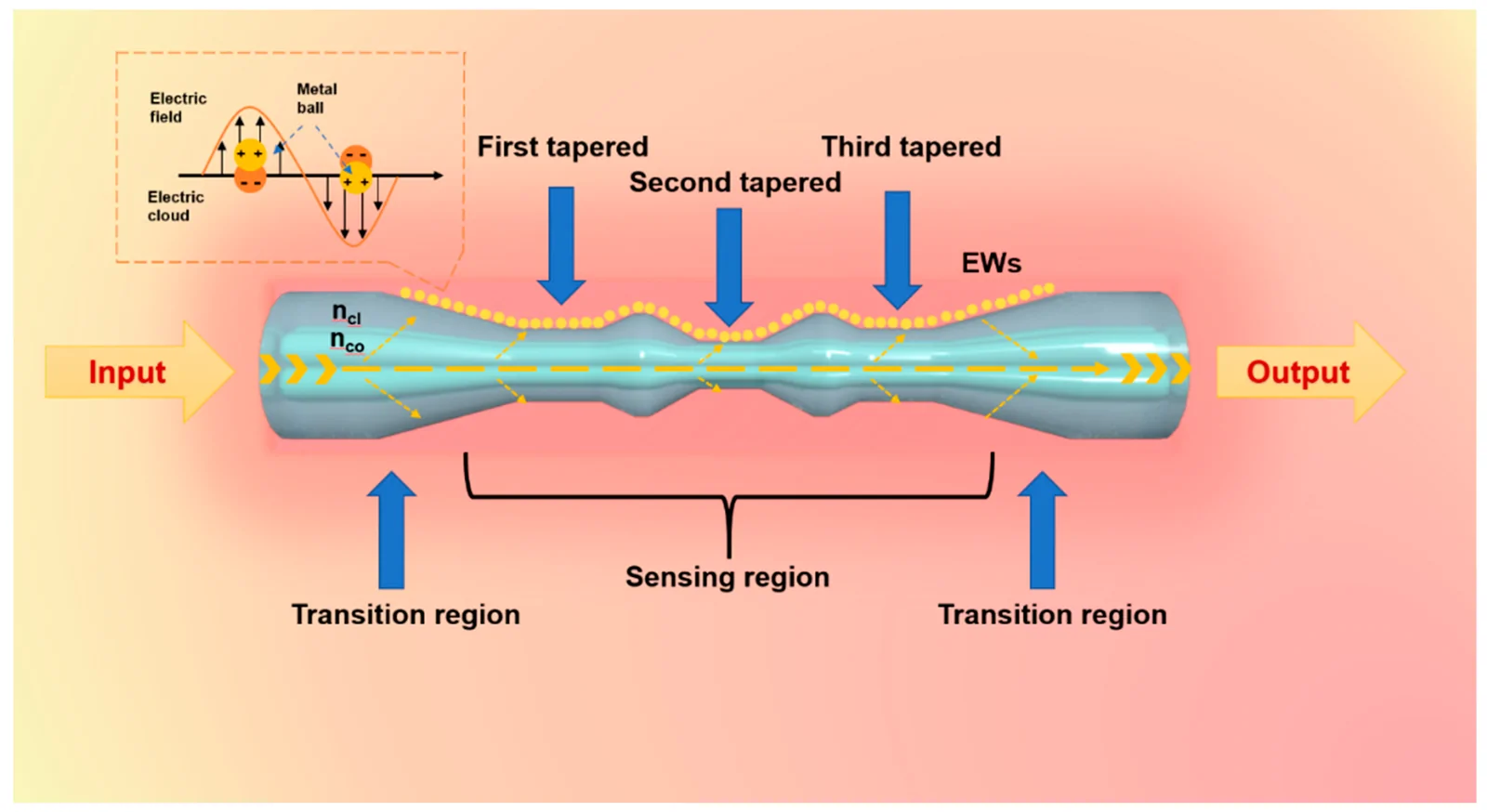
Working principle of the proposed biophotonic sensor.

The LSPR phenomenon exhibited by noble MNPs renders them highly suitable for biosensing applications. These sensors facilitate the detection of low-concentration biomolecules through precise monitoring of RI variations in the surrounding medium induced by target analytes. Enhanced sensing performance can be achieved through synergistic effects between MNPs and electromagnetic wave interactions, where an MNP-functionalized optical fiber surface generates amplified LSPR responses. The operational principle of LSPR-based sensors relies on spectral shifts in resonance wavelength or alterations in absorption peak intensity within the nanoparticles’ extinction spectrum, as shown in Figure 1. These optical changes directly correlate with RI perturbations caused by biomolecular interactions at the nanoscale interface. This detection mechanism capitalizes on biomolecules with dimensions comparable to noble MNPs, enabling effective surface interactions. A practical implementation involves the immobilization of mouse anti-triclosan monoclonal antibodies on nanoparticle surfaces, where specific binding events between the antibody and triclosan molecules in test samples induce measurable RI modifications. The resultant LSPR spectral variations provide quantitative information about the interaction between the sensing interface and the target analyte. Binding of triclosan to the immobilized antibody changes the local optical environment surrounding the AuNPs and consequently modifies the effective refractive index near the plasmonic surface. This local refractive index perturbation produces a measurable displacement of the LSPR-related transmission peak.

The relationship between the resonance wavelength shift and the local refractive index change can be described by the general LSPR sensing model reported in the literature [30]:

(1) $$\Delta \lambda =m\Delta n_{m}[1-e^{-\frac{2l}{d_{p}}}]$$

where (*m*) represents the nanoparticle refractive index sensitivity factor, (∆*n_m_*) represents the effective RI change induced by molecular adsorption, (*dp*) is the penetration depth of the evanescent field, and (*l*) represents the effective thickness of the molecular adsorption layer. These parameters describe the physical origin of the LSPR response and are dependent on the nanoparticle properties, optical field distribution, surrounding medium, and molecular adsorption layer.

In the present study, these parameters were not independently measured or used as separate fitting parameters to calculate the experimental calibration sensitivity. Instead, the equation is used to describe the physical mechanism by which molecular binding produces an optical wavelength shift. The quantitative sensing response was experimentally established by measuring the resonance wavelength at known triclosan concentrations and constructing the calibration relationship between the wavelength shift and triclosan concentration. Therefore, the experimentally obtained calibration slope represents the analytical sensitivity of the proposed biosensor, whereas the above equation provides the physical basis for understanding the refractive index-dependent LSPR response.

### 2.3. Simulation of Proposed Fiber Sensor Structure

The tapered MMF–FCF–MMF fiber configuration and the associated fusion-splicing and tapering procedures used in this work are based on established fiber-optic sensing and fabrication approaches reported in previous studies. Similar tapered MMF–FCF–MMF structures have been investigated for fiber-optic sensing, demonstrating that tapering can enhance the excitation of cladding modes and increase the interaction between the guided optical field and the surrounding medium. Tapered multimode-fiber structures have also been employed for evanescent-wave-based optical sensing. More recently, multi-core and four-core tapered fiber structures have been investigated for biophotonic sensing applications.

In the present study, these established fabrication principles were adapted to construct the specific MMF–FCF–MMF configuration used for triclosan detection. The FCF section was fusion-spliced to MMF segments at both ends and subsequently tapered using a combiner manufacturing system (CMS). The tapering parameters, including the electrode power, scanning velocity, and stage translation conditions, were experimentally optimized for the present sensing application. Therefore, the fabrication procedure should be regarded as an application-specific adaptation and optimization of established fiber-processing techniques rather than a completely new fiber-fabrication principle. The novelty of the present work lies primarily in the integration of this tapered fiber architecture with the AuNP/MWCNT/WS_2_-QD nanocomposite and triclosan-specific antibody recognition layer for label-free LSPR biosensing.

The fiber configuration and tapering strategy adopted in this work are based on well-established approaches in tapered and multicore fiber-optic biosensing [31]. It has been proved in previous studies that tapered and core-mismatched fiber structures can improve the evanescent field and allow the excitation of LSPR at the fiber surface [16,17]. Therefore, the basic principles of fiber tapering, fusion splicing, and evanescent-field enhancement used in the present study are based on established fiber-processing approaches rather than being entirely new fabrication principles.

In the present work, these established fiber-processing approaches were adapted to construct the specific MMF–FCF–MMF sensing structure. The FCF section was fusion-spliced to MMF segments at both ends and subsequently tapered using a combiner manufacturing system (CMS). The tapering parameters were experimentally optimized to obtain a suitable sensing geometry and evanescent-field interaction region for triclosan detection. Thus, the fabrication procedure represents an application-specific adaptation and optimization of established fiber-processing techniques.

**Figure 2:**
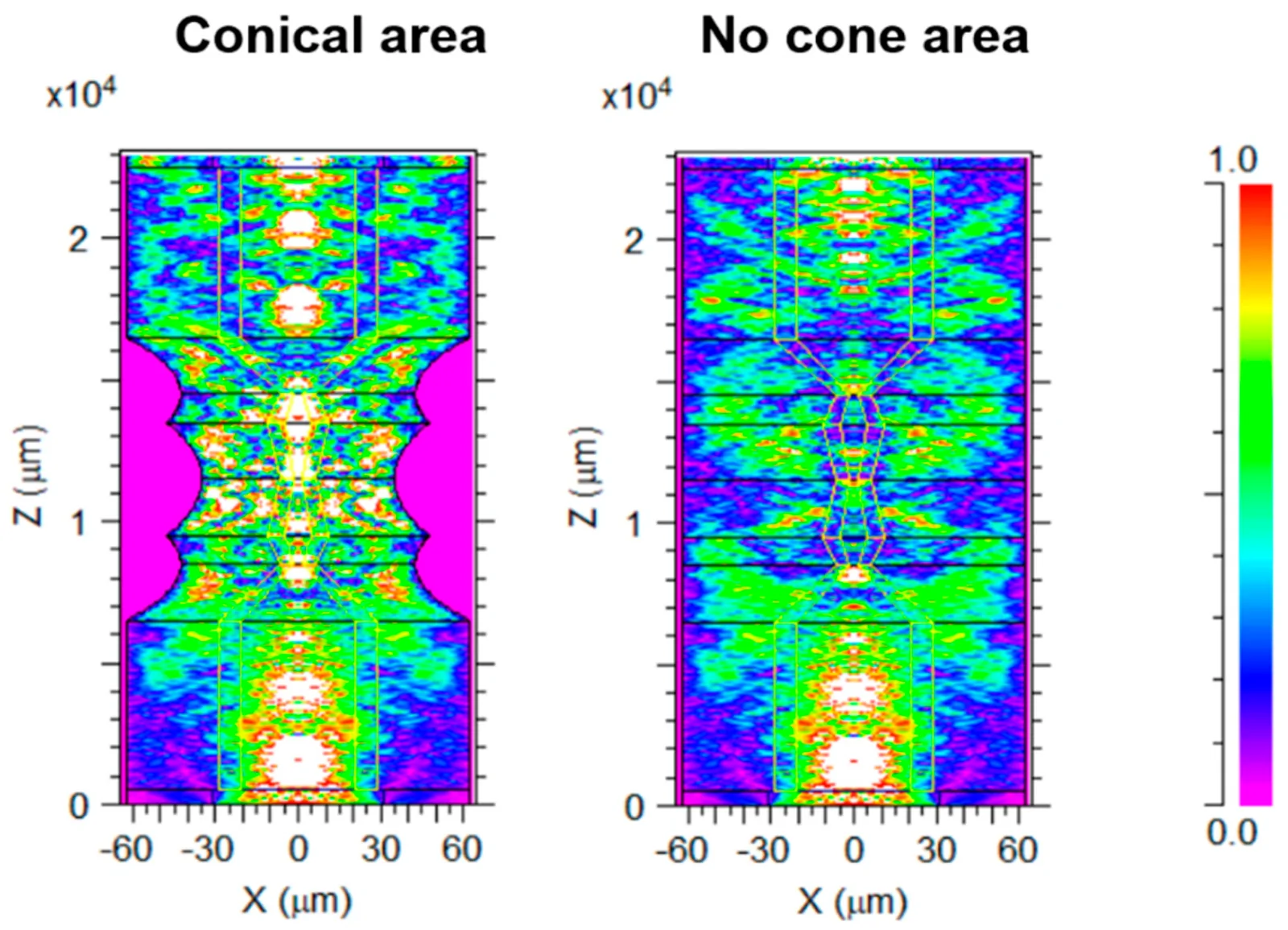
Simulation results of the proposed biophotonic fiber structure.

**Figure 3:**
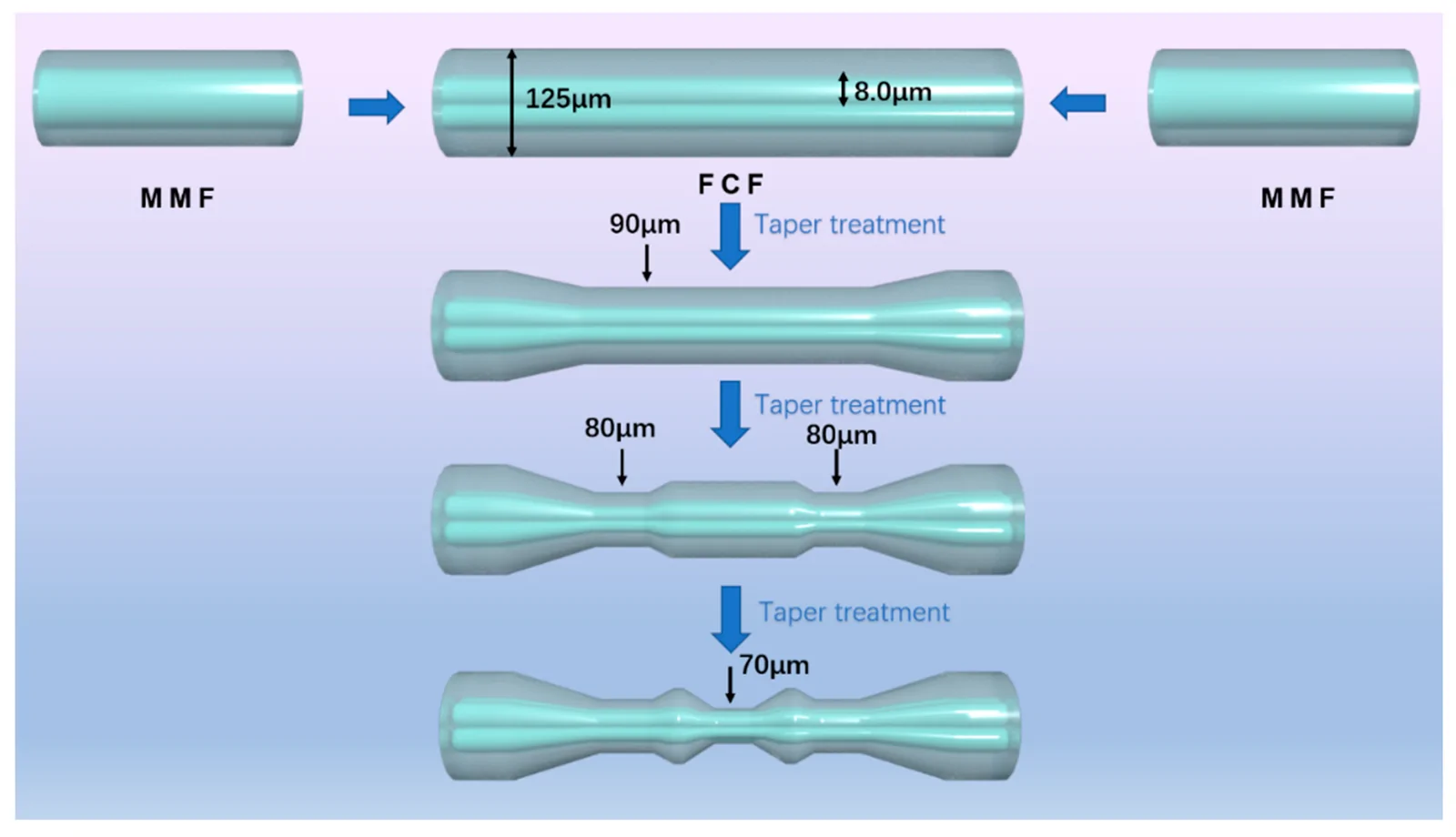
Schematic of fabrication process of the proposed fiber structure.

To further understand the evanescent field in optical fiber structures, the RSoft software (2018) was used to simulate the propagation of light in the optical fiber structures. The simulation results are shown in Figure 2. The simulation was conducted by splicing FCF with a core diameter of 8 µm and a cladding diameter of 125 µm and MMF with a core diameter of 62.5 µm and a cladding diameter of 125 µm. When light enters the tapered sensing area, the energy of the core mode significantly decreases, while that of the cladding mode significantly increases. Some of the core mode energy continues to propagate within the core, while some of the energy couples into the external medium to form EWs. Simulation results show that compared with non-tapered optical fibers, this structure can excite more EWs, thereby enhancing the LSPR effect. The proposed fiber-optic biosensor is fabricated through a sequential process involving fiber splicing and controlled tapering techniques. Initially, MMF segments are fusion-spliced to both ends of an FCF using a commercial fusion splicer. Prior to splicing, the polymer coatings of MMF and FCF are removed through mechanical stripping followed by ethanol cleaning of residual residues. The stripped fiber ends are precision-cleaned and cleaved with a fiber cleaver to ensure flat end faces, then aligned in the splicer’s electrode chamber for automated fusion under optimized discharge parameters. Following the MMF-FCF-MMF structural assembly, the CMS implements precise tapering of the FCF section. The CMS operates under near-vacuum conditions using a three-electrode configuration, enabling stable arc discharge with enhanced thermal stability compared to ambient-air splicing. This configuration facilitates precise control over the fiber’s conical profile during the drawing process. Critical tapering parameters including electrode power, scanning velocity, and stage translation speed are systematically optimized through iterative experiments to achieve high-precision fiber geometries with submicron repeatability. The tapering process employs three distinct programmatic stages: Program 1 reduces the fiber diameter from an initial 125 μm to a 90 μm waist diameter, Program 2 further tapers from 90 μm to 80 μm, and Program 3 achieves a final waist diameter of 70 μm. A multi-stage fabrication strategy is implemented where Program 1 first generates a single-taper profile in the MCF region, followed by Programs 2 and 3 superimposing additional tapers through coordinated axial displacement of the fiber stage. Figure 3 schematically illustrates this manufacturing workflow.

### 2.4. Synthesis of AuNPs, MWCNTs, and WS_2_-QDs

AuNPs were synthesized by the traditional Turkevich method [32], which is based on the reaction of HAuCl4 with the reducing agent trisodium citrate to prepare AuNPs. Specifically, 5 mg of MWCNTs powder was added to 10 mL of n, N-dimethylformamide (DMF) solution and ultrasonicated for 1 hour [33]. Additionally, 10 mg of WS_2_-QDs was added to 10 mL of DI water and ultrasonicated for 1 h [34].

### 2.5. Immobilization of AuNPs, MWCNTs, and WS_2_-QDs over Fiber Structure

The experimental protocol commenced with surface pretreatment of the fiber-optic probe through sequential purification processes. Initially, the probe underwent 20-minute immersion in an acetone solution followed by thorough rinsing with deionized water to achieve surface homogenization. Subsequently, the sensing region was treated with piranha solution (H_2_O_2_:H_2_SO_4_ = 3:7) for 30 minutes to oxidatively remove residual organic contaminants. After processing, the probe was carefully rinsed with deionized water and dried at 70 °C for 20 minutes. Then, the probe underwent a functionalization process, being immersed in a 1% 3-mercaptopropyl trimethoxysilane (MPTMS) ethanol solution for 12 hours to establish a monolayer of mercaptosilane as the interfacial coupling layer for subsequent nanoparticle immobilization [35]. After silanization, the probe was rinsed with ethanol to eliminate unreacted silane precursors, and then dried with nitrogen to ensure the complete removal of volatile components. For nanoparticle deposition, the functionalized probe was immersed in an AuNPs colloid suspension for 48 hours, then rinsed with ethanol to remove physically adsorbed NPs and dried with nitrogen to obtain a stable AuNPs-modified fiber surface [35]. This method facilitates the formation of a stable AuNP layer through covalent thiol–gold interactions, which is crucial for enhancing the sensing performance of the probe. Then, the AuNPs-coated area was immersed in the MWCNTs solution for 10 minutes and heated at 80 °C for 20 minutes. To ensure that the MWCNTs could effectively adhere to the probe surface, the above steps were repeated three times. Similarly, the probe was immersed in the WS_2_-QDs dispersion for 10 minutes and heated at 65 °C for 20 minutes. This procedure was repeated three times to ensure uniform deposition of WS_2_-QDs on the fiber surface. Repeat the above steps three times. It is worth mentioning that, as seen in the SEM images (Figure 9b,c), MWCNTs and WS2-QDs are deposited as a sparse network over the AuNPs layer rather than as a dense coating. This deposition configuration guarantees that a significant part of the surface of the AuNPs is exposed and available for subsequent MUA self-assembly and EDC/NHS activation. In addition, the network structure of the MWCNTs and WS_2_-QDs provides a high specific surface area, which improves the binding efficiency of target molecules. These three nanomaterials constitute a composite sensing interface that preserves the plasmonic activity of AuNPs and possesses a high loading capacity.

**Figure 4:**
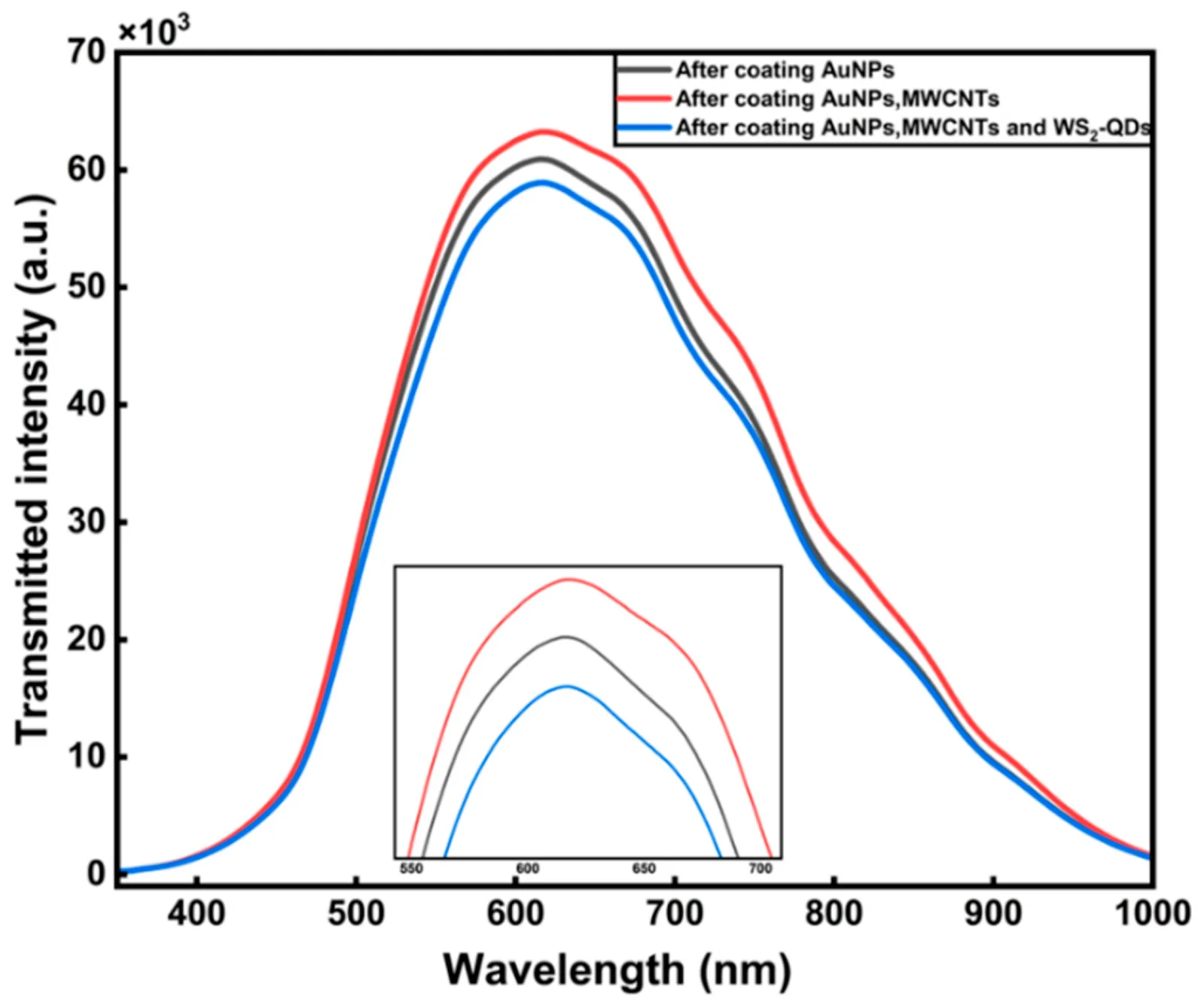
Comparison of transmitted intensity between the bare fiber probe and fibers with different functionalizations.

Additionally, the transmission intensity of the fiber-optic probes was compared before and after functionalization. As shown in Figure 4, the transmission intensity decreased after functionalization with AuNPs and NMs.

### 2.6. Antibody Functionalization over NMs-Immobilized Probe

**Figure 5:**
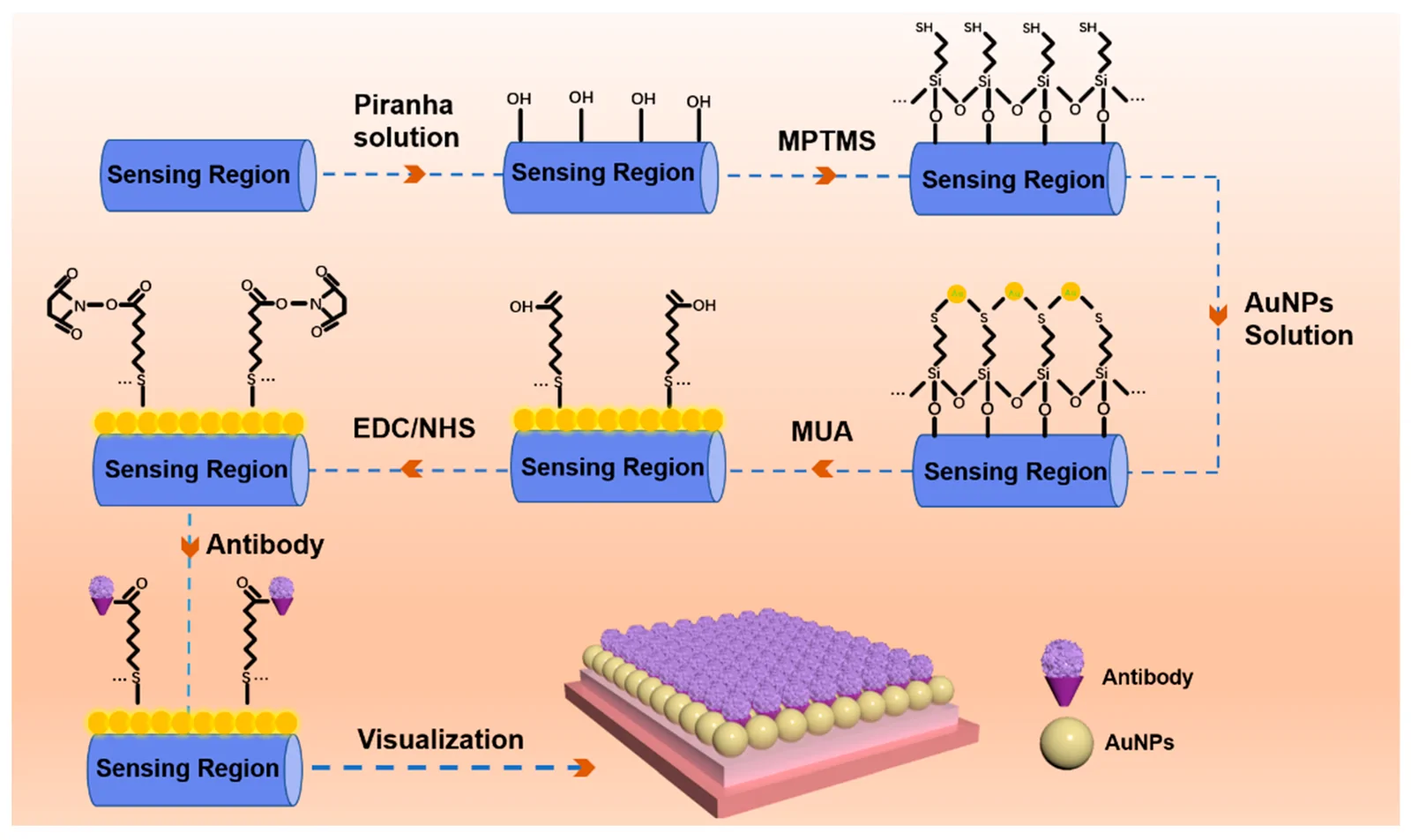
Schematic illustration of the surface functionalization and antibody immobilization process of the proposed fiber biosensor.

The functionalized optical fiber probe was further modified by the successive deposition of AuNPs, MWCNTs and WS_2_-QDs to covalently immobilize the antibody. The probe was first immersed in 10 mL of 0.5 mM 11-mercaptoundecanoic acid (MUA) in ethanol for 5 h to create a carboxyl-terminated self-assembled monolayer on the entire surface of the nanocomposite, which covered the AuNPs and the available active sites of the neighboring nanomaterials. Then, we performed the conjugation of the triclosan antibody using amine coupling chemistry through carbodiimide-mediated activation [31]. The probe was treated with 5 mL of an activation solution (containing 50 mM N-hydroxysuccinimide (NHS) and 200 mM 1-ethyl-3-(3-dimethylaminopropyl) carbodiimide (EDC)) for 30 min to activate the surface carboxyl groups. In this functionalization sequence, MPTMS and EDC/NHS serve different chemical functions. MPTMS introduces thiol (–SH) groups onto the hydroxylated fiber surface, which subsequently enables AuNP immobilization through Au–S interactions. After AuNP deposition, MUA provides terminal carboxyl (–COOH) groups on the nanocomposite surface. EDC/NHS then activates these carboxyl groups by forming an NHS-activated intermediate, which can react with primary amino groups of the antibody to form stable amide bonds. Therefore, EDC/NHS does not activate the thiol groups introduced by MPTMS; rather, it activates the carboxyl groups introduced by MUA for covalent antibody immobilization. The activated probe was rinsed thoroughly with deionized water and then incubated in an antibody solution at 4 °C for 2 h to favor covalent immobilization. After incubation, nonspecific binding sites were blocked by incubation with a 1% skim milk solution (100 mg milk powder was dissolved in 10 mL DI water) for 10 min. This immobilization strategy employs a random covalent coupling mechanism by means of the carboxyl–amine reaction, rather than oriented antibody immobilization. However, this protocol provides stable binding of the antibodies on the nanocomposite surface because of the numerous binding sites available and retains their specific molecular recognition ability for the target analyte. Figure 5 schematically shows the simple immobilization process.

The antibody was covalently immobilized onto the functionalized nanocomposite surface through the EDC/NHS-mediated coupling reaction. In contrast, the subsequent recognition of triclosan by the immobilized antibody occurs through non-covalent molecular interactions, including hydrogen bonding, hydrophobic interactions, and other complementary intermolecular interactions within the antibody-binding site. Therefore, the antibody remains covalently attached to the sensing interface, whereas triclosan binding is reversible and non-covalent. This distinction is important for the subsequent solvent-assisted washing step, which is intended to remove the bound analyte while retaining the immobilized antibody on the sensing surface.

### 2.7. Preparation of Triclosan Detection

A stock solution of triclosan was prepared at a concentration of 10 mM by dissolving 57.9 mg of TCS in 20 mL of DMF. The triclosan stock solution was diluted with PBS to prepare working solutions with triclosan concentrations of 0, 0.1,1,10, 20, 25, 30, 35 and 40 μM for sensing experiments. All working solutions contained 2% (*v*/*v*) of the final DMF content, with PBS as the main solvent, to minimize the influence of DMF on the sensing results.

### 2.8. Experimental Setup for Triclosan Detection

**Figure 6:**
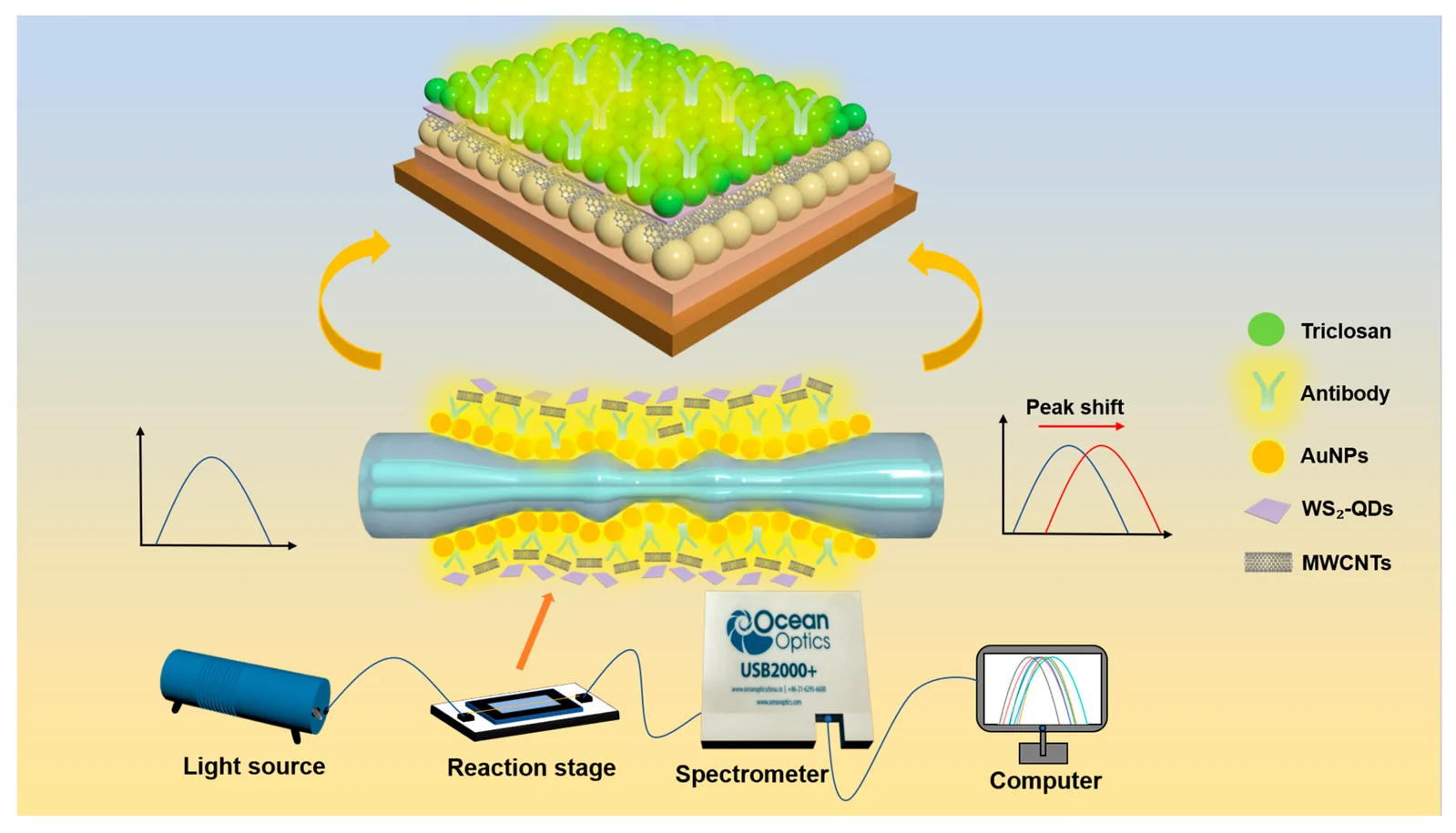
Experimental setup for triclosan measurement.

The prepared triclosan working solutions in PBS were used for the calibration measurements. The experimental setup employed a tungsten–halogen lamp to generate LSPR excitation on the functionalized fiber surface. A synchronized data acquisition interface linked the spectrometer to computational analysis software for real-time spectral monitoring. To minimize mechanical perturbations during measurements, the biosensor probe was securely mounted in a customized V-groove alignment fixture equipped with a strain-relief mechanism, ensuring that the tapered region remained straight and free from axial tension throughout the measurements. The fiber ends were fusion-spliced to the light source and spectrometer to avoid connector-induced stress fluctuations. The alignment fixture was placed on an optical table with active vibration isolation to suppress environmental disturbances. All experiments were conducted in a temperature-controlled laboratory, with the ambient temperature maintained at 25 ± 0.5 °C. Prior to each measurement, the triclosan solutions were equilibrated at room temperature for 30 minutes to ensure thermal equilibrium with the sensing system. The concentration of triclosan was determined using the experimental setup shown in Figure 6. Systematic measurements were conducted using triclosan solutions across nine concentration levels to establish a quantitative detection model. Spectral analysis revealed a concentration-dependent redshift in the transmission intensity peak wavelength, with the magnitude of the shift exhibiting a linear correlation to analyte concentration. This wavelength modulation mechanism enabled precise triclosan quantification through calibration of peak wavelength displacement against established concentration standards, demonstrating the biosensor’s capability for LSPR-based triclosan detection. The optical interrogation system consisted of a tungsten-halogen illumination source (Ocean Optics HL-2000, FL, USA) coupled to an Ocean Optics USB2000+ spectrometer for transmission-spectrum acquisition. The USB2000+ spectrometer has a specified wavelength resolution of approximately 0.02 nm, which is sufficient to resolve the resonance-wavelength shifts observed in the investigated concentration range. A synchronized data acquisition interface was used for real-time spectral monitoring and data analysis.

Because the anti-triclosan antibody was covalently immobilized on the MUA-functionalized nanocomposite surface through EDC/NHS-mediated amide coupling, whereas the subsequent antibody–triclosan recognition occurs through reversible non-covalent interactions, the bound triclosan can be removed from the antibody recognition sites without intentionally disrupting the covalent linkage between the antibody and the sensing interface. In the regeneration experiments, DMF was used to rinse the sensing probe repeatedly to facilitate the removal of bound triclosan, followed by subsequent sensing measurements under the same experimental conditions. This regeneration procedure was experimentally evaluated over three reuse cycles at triclosan concentrations of 10 and 40 μM.

## 3. Results and Discussion

### 3.1. Optimization of Fiber Structure

**Figure 7:**
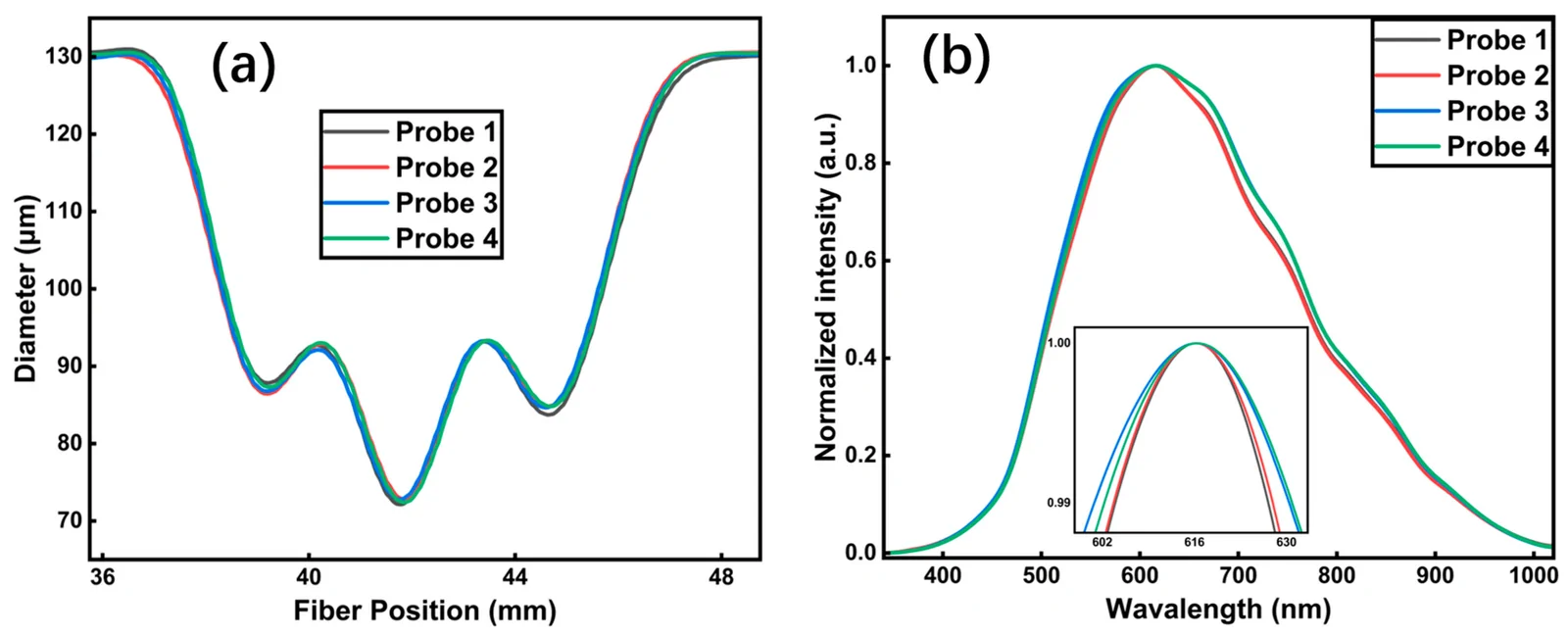
(**a**) Diameter scan of fabricated biophotonic sensor structure, (**b**) transmission spectrum of fabricated optical fiber structure.

The CMS has emerged as a preferred technology for fabricating optical fiber probes due to its significant advantages compared to conventional hydrogen–oxygen flame heating methods, particularly in terms of superior efficiency, reproducibility, and operational stability. This advanced system enables the formation of tapered fiber structures through precisely controlled arc discharge processes, where a three-electrode arc platform applies programmed movement tension to gradually stretch the FCF into tapered geometries. Critical geometric parameters can be systematically adjusted and refined through iterative optimization protocols to achieve target specifications. As illustrated in Figure 7a, CMS scanning characterization reveals that the fabricated fibers exhibit uniform waist diameters with smooth transitional profiles, confirming process consistency. While reduced waist diameters enhance biosensor sensitivity, excessively thin tapered regions demonstrate mechanical fragility during functionalization or measurement applications. Figure 7b presents the transmission spectra of the fabricated fiber-optic biosensors, showing consistent initial peak positions across multiple probes. This spectral alignment confirms the high repeatability of the CMS-based manufacturing process, which is essential for reliable biosensor production. The combined structural and optical analyses demonstrate that CMS technology effectively balances sensitivity requirements with mechanical robustness through controlled taper dimension optimization.

### 3.2. Characterization of Used Nanomaterials

**Figure 8:**
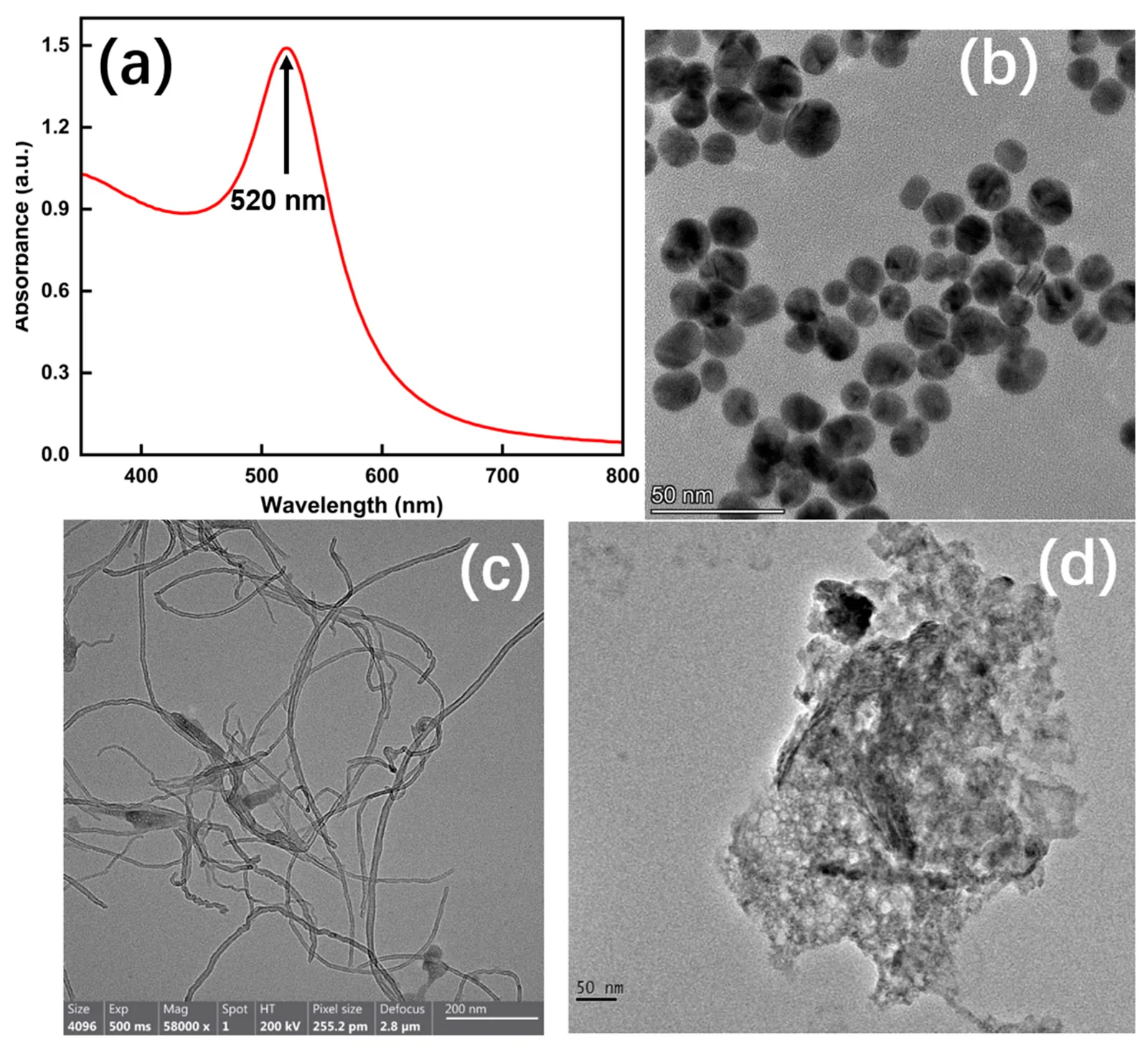
(**a**) Absorbance spectrum of the AuNPs. TEM images of (**b**) AuNPs, (**c**) MWCNTs, and (**d**) WS_2_-QDs. (**b**) shows that AuNPs present a uniform 10 nm size on the fiber surface, and (**c**) shows that the MWCNTs exhibit a characteristic filamentous morphology. (**d**) shows that the WS_2_-QDs have a layered structure, which amplifies the fiber’s effective surface area to enhance antibody immobilization capacity. These morphological analyses verify the controlled synthesis and functional integration of the NMs critical for biosensor optimization.

**Figure 9:**
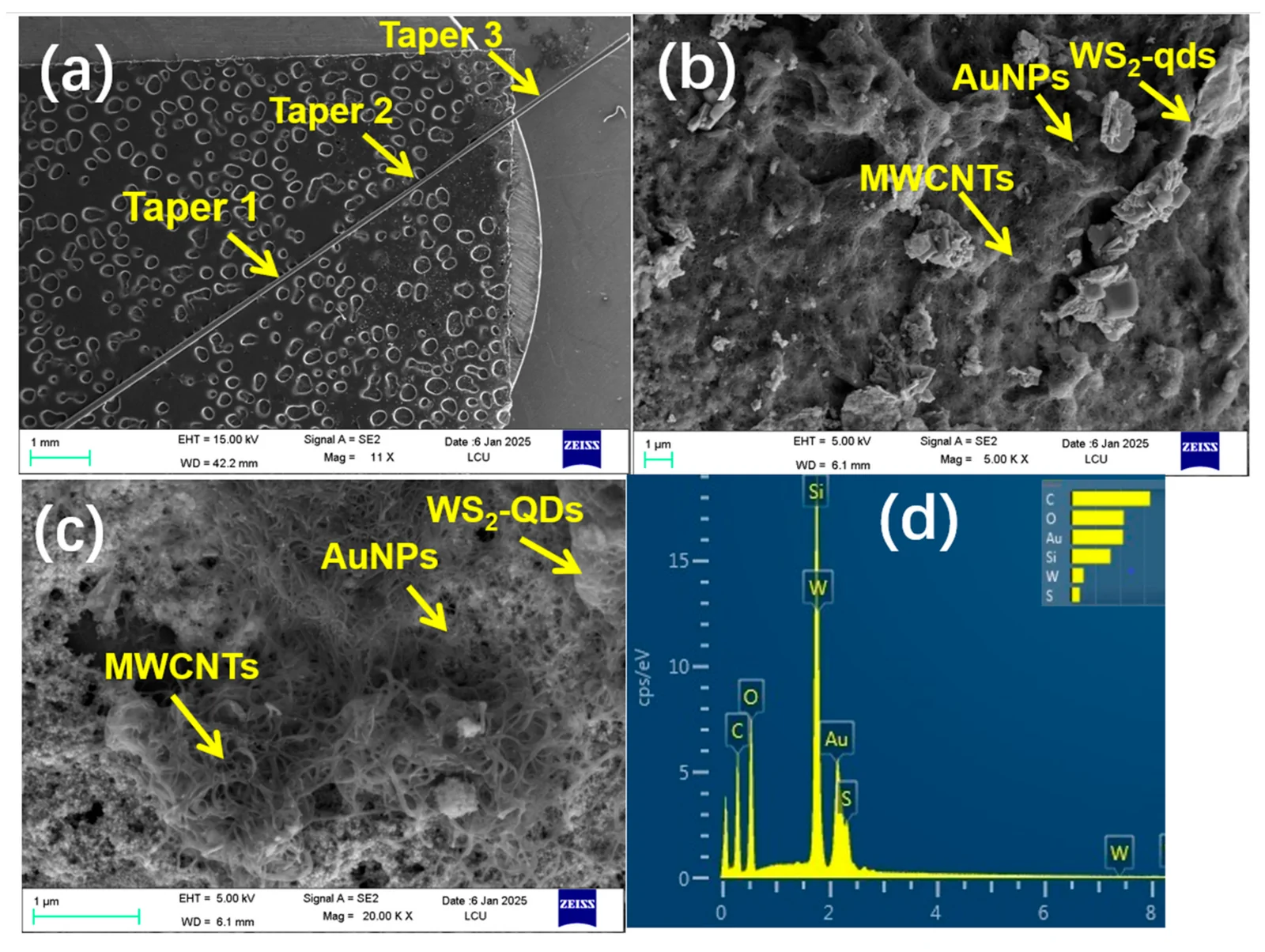
(**a**) SEM image of optical fiber surface; (**b**,**c**) SEM images of nanoparticles on optical fiber surface; and (**d**) EDS analysis of sample shown in (**c**).

The morphology and dimensions of the AuNPs critically influence sensing performance. The ultraviolet-visible spectral analysis shown in Figure 8a confirmed successful AuNPs synthesis, evidenced by a characteristic absorption peak at 520 nm corresponding to spherical NPs with an average diameter of 10 nm. High-resolution TEM characterization further revealed the structural details of the nanocomponents. The LSPR peak position is not solely dependent on size but is also influenced by the refractive index of the surrounding medium, the particle aggregation state, and the presence of stabilizing agents (citrate in this case). Furthermore, the EDS elemental analysis in Figure 9d confirms the presence of W and S elements, providing additional evidence for the successful immobilization of WS_2_-QDs within the composite sensing layer.

### 3.3. Characterization of NPs-Coated Biosensor Structure

The fiber sensing region was functionalized with AuNPs, MWCNTs, and WS_2_-QDs. SEM imaging provided high-depth-of-field visualization of the biosensor surface, enabling comprehensive analysis of the NM distribution and surface morphology. The SEM images of the NPs-modified fiber sensing area are shown in Figure 9a–c, which show the MWCNT and WS_2_-QD structures assembled and immobilized on the fiber substrate. Figure 9d shows the compositional verification through SEM-EDS elemental mapping, which identified characteristic Au, C, W, and S signatures, confirming the successful deposition of all three nanomaterial components. This analytical approach enables a quantitative assessment of the surface chemical composition, validating the functionalization process critical for biosensor performance optimization.

### 3.4. Sensing Results

**Figure 10:**
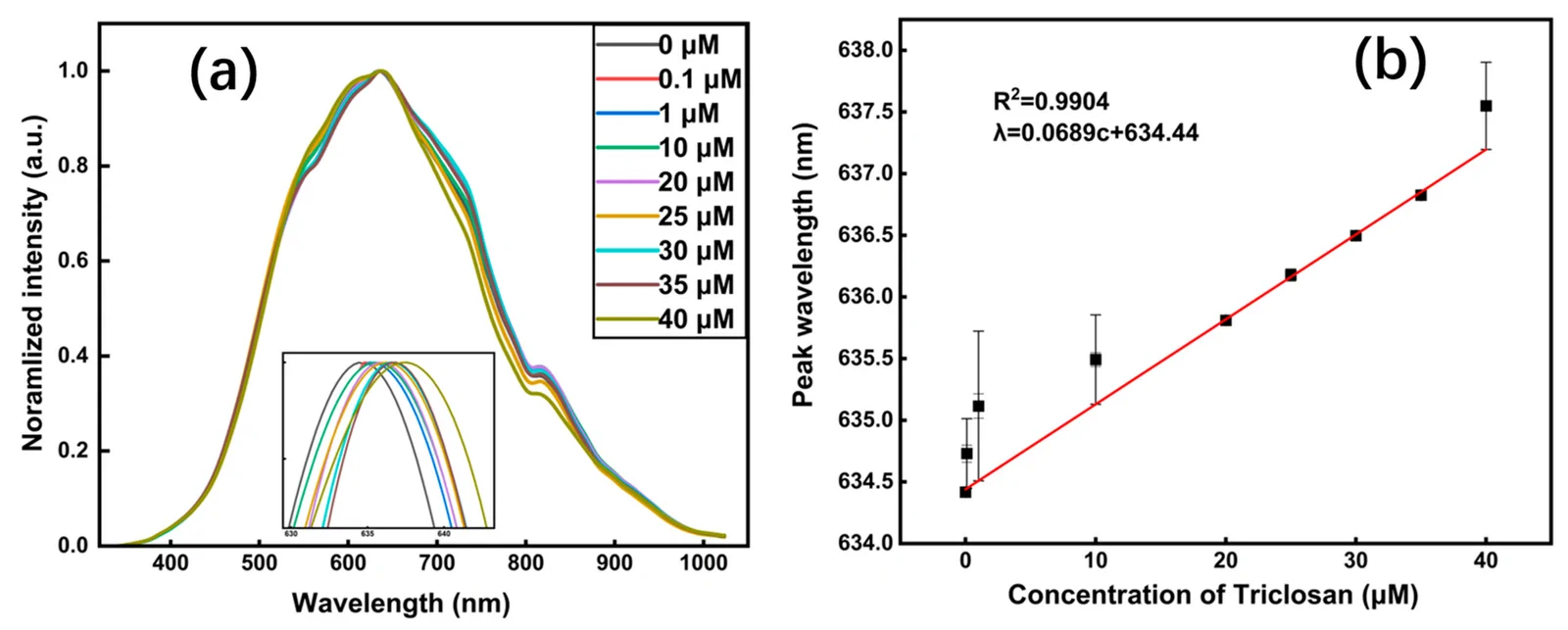
(**a**) Sensing spectrum, (**b**) Linear calibration curve of the proposed biosensor for triclosan detection. The data are presented as mean ± SD (n = 3).

The biosensor detection performance was evaluated by the determination of triclosan in the concentration range of 0–40 µM through sequential detection from low to high concentrations. The measurements were performed using three individually fabricated biosensor probes under the same experimental conditions. The resonance wavelength of the transmission spectrum was gradually red-shifted with increasing triclosan concentration, as shown in Figure 10a. The analyte concentration was quantified by following the spectral peaks since the wavelength shift was directly proportional to the analyte concentration. Sensitivity was selected as a key performance metric, defined as the slope of the linear regression of the calibration data. A good linear relationship between the peak wavelength and triclosan concentration in the range of 0–40 µM is shown in Figure 10b with an R^2^ value of 0.9904. The calibration data are presented as mean ± standard deviation (SD), and the corresponding SD error bars are shown in Figure 10b to represent the variability among independently fabricated biosensor probes. The sensitivity of the proposed biosensor was calculated as 0.0689 nm/µM. The linearity established between the resonance wavelength and triclosan concentration shows the quantitative detection capability of the biosensor proposed here. The correct equation is

*λ* = 0.0689*c* + 634.44 (2)

where the peak wavelength of the LSPR spectrum is represented by *λ*, while the concentration of the triclosan solution is denoted as *c*. The limit of detection (LoD) refers to the lowest concentration of the target analyte that can be reliably detected by the biosensor, which is another important indicator to measure the performance of the biosensor, and the formula is expressed as:

(3) $$\mathrm{LoD}=\frac{3\times \mathrm{SD}}{\mathrm{Sensitibity}}$$

The triclosan biosensor has an LoD of 3.87 µM.

Additionally, to further clarify the contribution of the functional nanomaterials to the sensing performance, control experiments were performed using probes with different functionalization configurations. The sensitivity and LoD obtained from these control experiments are summarized in Table 1. Compared with the AuNP-only probe, the incorporation of MWCNTs and/or WS_2_-QDs resulted in changes in the sensing performance, while the AuNPs/MWCNTs/WS_2_-QDs composite exhibited the highest sensitivity and lowest LoD among the tested configurations. These results indicate that the incorporation of MWCNTs and WS_2_-QDs contributes to the enhanced sensing response of the composite-functionalized probe. The improved performance can be attributed to the complementary functional characteristics of the nanocomposite, which facilitate the interaction between the optical field and the sensing interface. The experimentally demonstrated performance enhancement also supports the rationale for integrating the three nanomaterials with the tapered fiber structure to construct the proposed sensing platform.

### 3.5. Reproducibility and Repeatability Test

**Figure 11:**
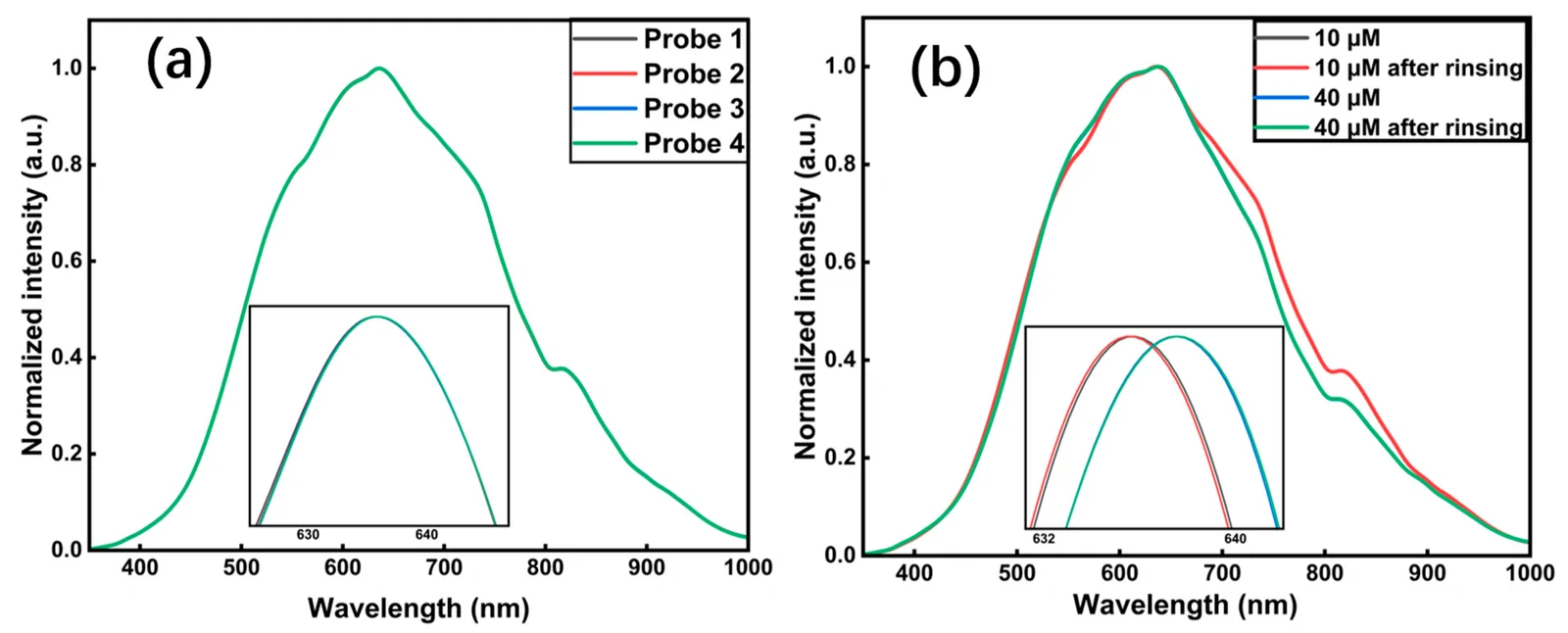

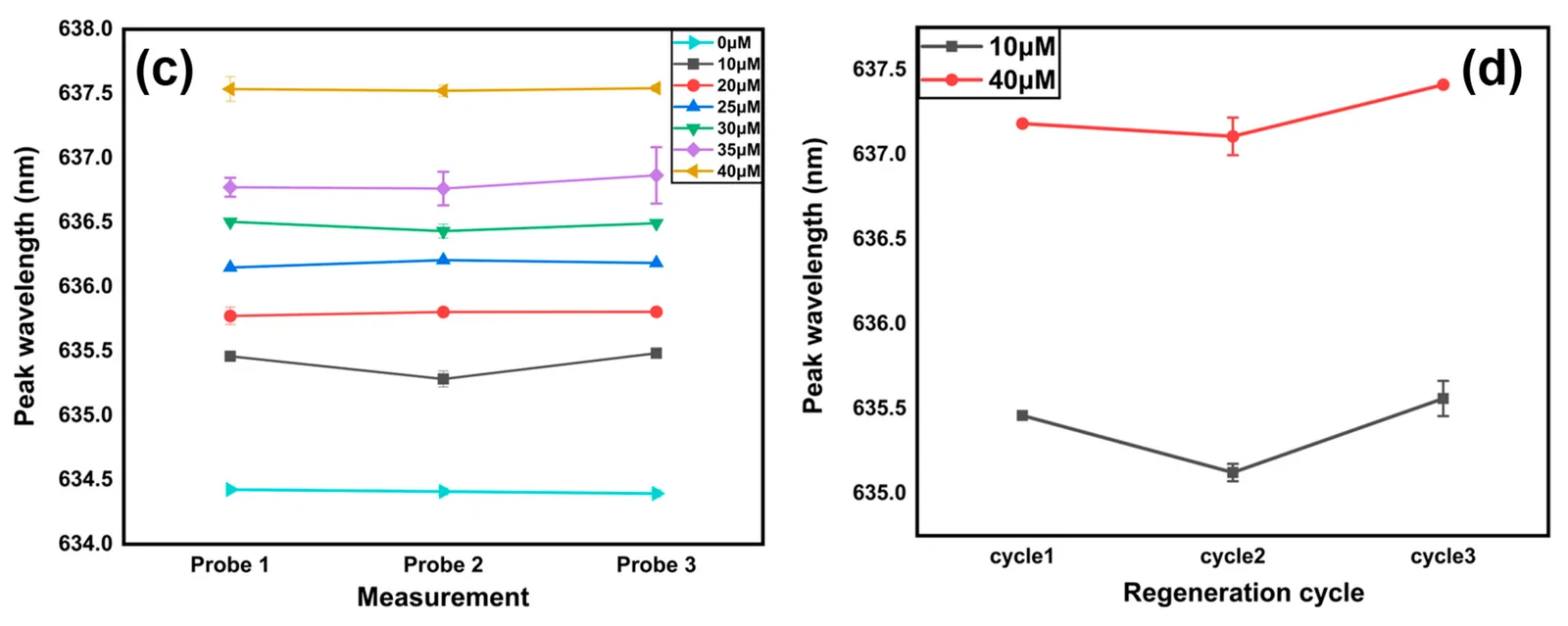
(**a**) Reproducibility and (**b**) reusability test of proposed biosensor; (**c**) Reproducibility of the sensor using three independently fabricated probes. Error bars represent SD; and (**d**) reusability of the sensor over three cycles at 10 and 40 μM triclosan. Error bars represent SD.

Reproducibility, defined as the ability to obtain consistent peak wavelength measurements when detecting identical analyte concentrations across multiple trials using biosensors from the same batch, was experimentally verified. As illustrated in Figure 11a, two distinct biosensors were employed to detect 40 µM triclosan solutions, demonstrating closely aligned peak wavelength values with minimal variation. This high degree of measurement consistency confirms the good reproducibility of the fabricated sensing devices. To further assess the reproducibility of the proposed biosensor, three independently fabricated biosensor probes were evaluated under identical experimental conditions. For this independent-probe reproducibility evaluation, the probes were tested at seven selected triclosan concentrations (0, 10, 20, 25, 30, 35, and 40 μM), and the corresponding peak wavelength responses were recorded. The measurements were repeated, and the standard deviation (SD) was calculated at each concentration. The resulting error bars provide a quantitative evaluation of the variability among the independently fabricated probes. As shown in Figure 11c, the biosensor responses exhibited relatively small variations, indicating good consistency among the independently fabricated probes.

The reusability and regeneration of the proposed biosensor were studied by consecutive detection of the triclosan solutions at 10 and 40 μM using the same biosensor unit. After each sensing measurement, the sensor probe was rinsed repeatedly with DMF to remove the bound triclosan from the functionalized sensing interface, and the subsequent sensing measurement was performed (Figure 11b). Three cycles of regeneration/reuse were carried out at both concentrations. The peak wavelength responses were relatively consistent over three cycles, as shown in Figure 11d. At a triclosan concentration of 10 μM, the peak wavelength values were around 635.5, 635.1 and 635.6 nm for cycles 1-3, respectively, and the corresponding values at a triclosan concentration of 40 μM were around 637.2, 637.1 and 637.4 nm. Small differences between the cycles, as seen from the SD error bars, pointed to an invariant spectral response of the biosensor after multiple regeneration and reuse cycles. These results support the repeated-use performance of the proposed biosensor under the investigated conditions.

### 3.6. Stability and pH Test

**Figure 12:**
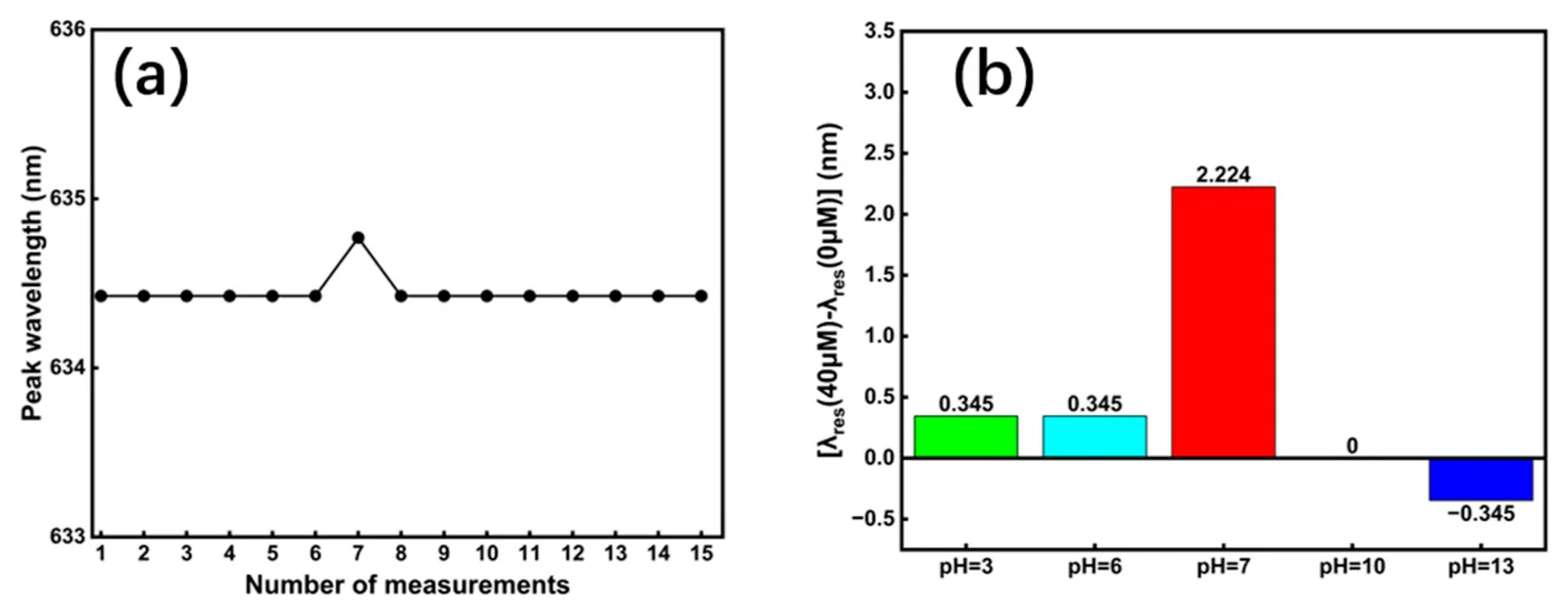
(**a**) Stability and (**b**) pH test of proposed biosensor.

Stability assessment serves as a critical evaluation method for determining biosensor operational consistency over extended durations. This methodology involves repeated measurements of biosensor signal outputs to quantify temporal performance variations. Experimental validation was performed through successive spectral acquisitions fifteen times in dimethylformamide (DMF) medium, with systematic documentation of wavelength-specific responses. Figure 12a shows the variation in peak wavelength across 15 measurement cycles. Quantitative analysis revealed a minimal standard deviation (SD) of 0.089, demonstrating exceptional signal reproducibility. This SD of 0.089 nm was calculated from 15 consecutive baseline (0 µM) measurements under identical conditions. The low SD value directly confirms the biosensor’s capability to maintain measurement precision under prolonged operational conditions.

Solution reactions often require specific pH conditions. pH testing identifies the optimal environment for the analyte, ensuring that reactions proceed effectively. Triclosan solutions (0 and 40 µM) were tested at pH 3, 6, 7, 10, and 13. At each pH, the peak wavelength difference between low and high triclosan concentrations was analyzed. As shown in Figure 12b, the greatest peak wavelength shift occurred at pH 7, demonstrating that this environment is optimal for the experiment. The observed maximum biosensor response at pH 7 can be attributed to the optimal conditions for antibody–antigen binding. The anti-triclosan antibody, like most proteins, retains its full biological activity and structural integrity at physiological pH. Furthermore, triclosan (pKa ≈ 7.8–8.1) exists in a molecular state at pH 7 that is most favorable for specific recognition by the antibody’s binding site [36]. Extreme pH values may lead to partial antibody denaturation or alter the charge state of triclosan, thereby reducing binding efficiency and the resultant LSPR shift.

### 3.7. Selectivity Test

**Figure 13:**
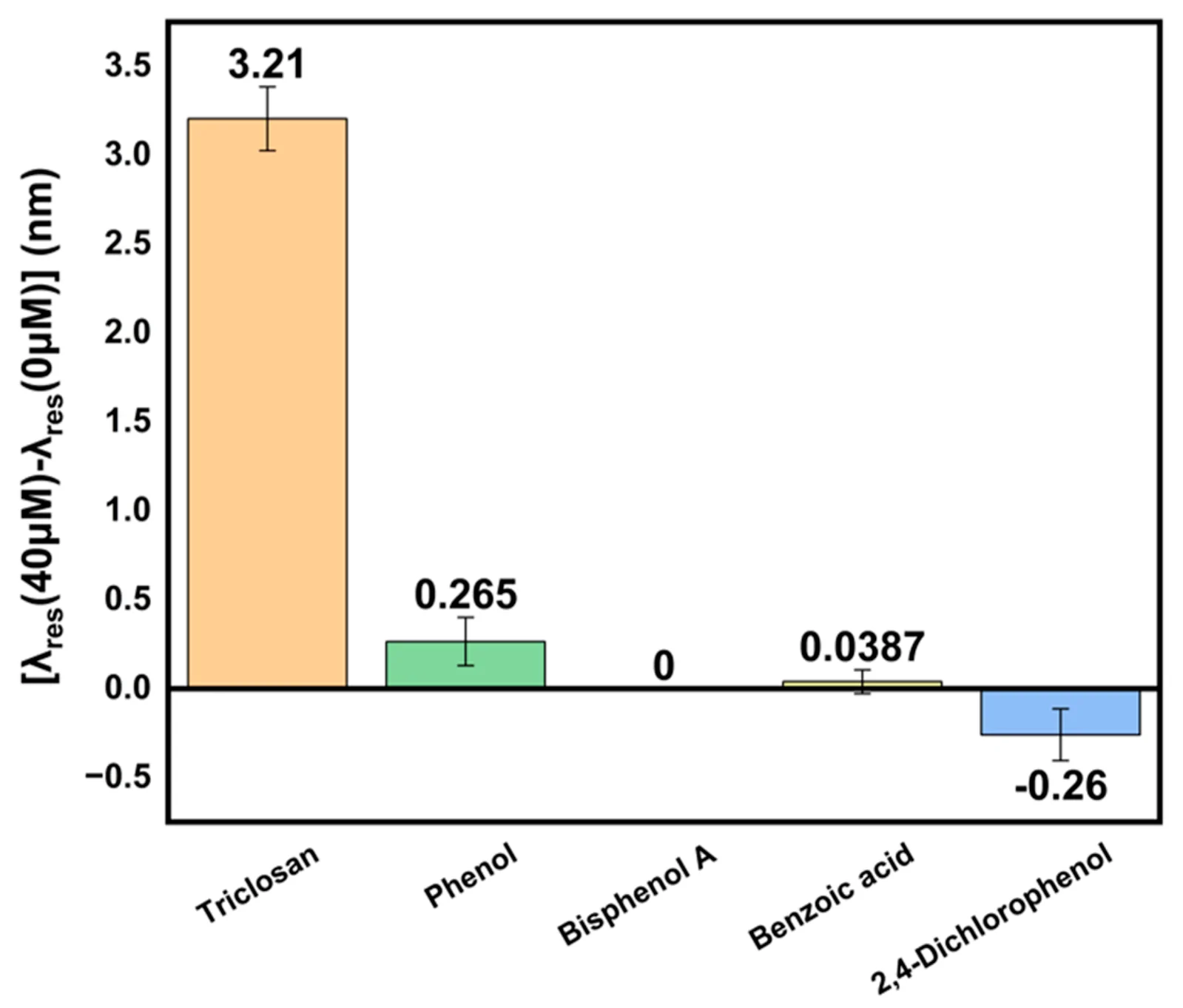
Selectivity test of the proposed biosensor. The results are presented as mean ± SD (n = 3).

The selectivity of the proposed biosensor was investigated in the presence of phenol, bisphenol A, benzoic acid, and 2,4-dichlorophenol as possible interfering substances. The interfering compounds were not mixed in a single test solution; instead, each compound was prepared and measured separately under the same experimental conditions. The measurements were performed in triplicate (n = 3) and the results are depicted as the mean ± SD in Figure 13. The measured interfering substances caused considerably lower shifts in the wavelength than the response to triclosan, which shows a preferential response of the biosensor to triclosan under the investigated conditions.

Exposure to 2,4-dichlorophenol led to a decrease in the resonance wavelength compared with the blank, which represents a negative shift in wavelength. This behavior could be ascribed to its different interaction with the functionalized sensing interface compared with triclosan. The relatively low responses of the tested interfering compounds may be explained by differences in their molecular frameworks and the resulting molecular complementarity with the antibody-binding site. The immobilized anti-triclosan antibody recognizes the complete molecular skeleton, the spatial arrangement, and the specific functional groups of the target molecule. Triclosan has a typical chlorinated diphenyl ether structure with a phenolic hydroxyl group, but phenol, bisphenol A, benzoic acid, and 2,4-dichlorophenol do not share the same general structural features. Some of these compounds bear functional groups or aromatic moieties in common with triclosan, but their overall molecular makeup and spatial arrangements differ from those of the target molecule, leading to much weaker recognition by the immobilized antibody under the test conditions.

For example, 2,4-dichlorophenol is similar to triclosan in that it has a phenolic hydroxyl group and chlorine substituents, but it lacks the second aromatic ring and ether linkage present in triclosan. These structural differences may account for its different response pattern and the negative wavelength shift in Figure 13. However, the response pattern of 2,4-dichlorophenol was still distinguishable from that of triclosan, confirming the preferential selectivity of the proposed biosensor under the tested conditions.

### 3.8. Testing of Real Samples

In practical applications, evaluating biosensor performance using real samples is important because sample matrices may affect the sensing response. Triclosan is commonly used as an antibacterial agent in personal care products. Therefore, toothpaste and facial cleanser were selected as representative real samples to evaluate the applicability of the proposed biosensor.

A 2 g portion of each product was dissolved in PBS, followed by centrifugation for 1 h, and the supernatant was collected for subsequent measurements. Both unspiked samples and samples spiked with triclosan at 20 and 40 μM were analyzed. The unspiked samples were used to characterize the background response of the corresponding sample matrices, whereas the spiked samples were used for recovery evaluation at two different concentration levels. The results are summarized in Table 2.

For toothpaste, the recovery rates were 87–109% at 20 μM and 80–102% at 40 μM, with RSD values of 10.1% and 11.3%, respectively. For the facial cleanser, the recovery rates were 89–95% at 20 μM and 91–114% at 40 μM, with RSD values of 9.4% and 13.1%, respectively. These results demonstrate that the proposed biosensor can generate reproducible responses to triclosan in the tested toothpaste and facial cleanser matrices over the investigated spiked levels.

### 3.9. Comparison with Existing Biosensors

Table 3 presents the performance characteristics of various analytical methods for triclosan detection, including electrochemical, activated carbon-based, fluorescence, surface-enhanced Raman scattering (SERS), surface plasmon resonance (SPR), and LSPR-based approaches. While existing detection methods, such as electrochemical techniques and activated carbon composite materials, have achieved significant progress, recent optical and plasmonic approaches have also demonstrated the feasibility of sensitive and rapid triclosan detection. These methods employ different sensing mechanisms, including molecular recognition, fluorescence responses, and plasmon-enhanced optical signals, providing alternative strategies for triclosan analysis. In contrast, the proposed biosensor combines a tapered fiber-optic configuration with a functional nanocomposite sensing interface, providing simple operation, a wide linear range, and potential resistance to matrix interference.

As shown in Table 3, optical and plasmonic methods have provided promising alternatives to conventional analytical techniques for triclosan detection. SPR-based sensors have demonstrated the feasibility of molecularly imprinted optical recognition, while SERS-based approaches have enabled rapid detection using plasmonic nanostructures. More recent fluorescence and SERS-based methods have further improved detection capability and facilitated applications in environmental and complex samples. However, these approaches generally rely on planar SPR substrates, Raman-active nanostructures, or fluorescence-based sensing interfaces. In comparison, the integration of a tapered fiber-optic structure with a multifunctional ternary nanocomposite and antibody-based recognition provides a different strategy for LSPR-based triclosan sensing.

The novelty of the proposed biosensor is further supported by the comparative experiments presented in Section 3.4. Rather than relying on a single nanomaterial, the proposed sensing interface integrates AuNPs, MWCNTs, and WS_2_-QDs with the tapered fiber structure. The control experiments demonstrate a progressive improvement in analytical performance from the AuNPs-only configuration to the binary and ternary nanocomposite configurations. In particular, the AuNPs/MWCNTs/WS_2_-QDs composite achieved a sensitivity of 0.0689 nm/μM and an LoD of 3.87 μM, outperforming the corresponding AuNPs-only, AuNPs/MWCNTs, and AuNPs/WS_2_-QDs configurations. These experimental results provide direct evidence that the integrated nanocomposite design contributes to the enhanced sensing performance of the proposed fiber-optic platform.

## 4. Conclusions

The core innovation of this research lies in the construction of a multi-element collaborative sensing platform that integrates a tapered fiber-optic structure with a functionally integrated plasmonic nanocomposite, providing new design concepts and an experimental basis for the development of high-performance fiber-optic biosensors for environmental monitoring. Control experiments with different nanomaterial configurations further demonstrated the contributions of MWCNTs and WS_2_-QDs to the sensing performance. The AuNPs/MWCNTs/WS_2_-QDs ternary nanocomposite achieved the highest sensitivity of 0.0689 nm/μM and the lowest LoD of 3.87μM among the tested configurations, providing direct experimental support for the proposed multi-component sensing interface. The tapered fiber-optic probe facilitates sufficient evanescent-field generation to enhance LSPR excitation. The sensitivity of the proposed biosensor was augmented through functionalization with AuNPs, WS_2_-QDs, and MWCNTs, while specificity was conferred by the immobilized triclosan antibodies. Experimental results demonstrated a linear correlation (R^2^ = 0.9904) between the resonance wavelength shift and triclosan concentration within the 0–40 μM range, with a calculated LoD of 3.87 μM. Comprehensive evaluation confirmed acceptable reproducibility, repeatability, stability, pH test, and selectivity. Real-sample validation collectively substantiates the biosensor’s practical utility. Future work will focus on lowering the LoD, enhancing sensitivity, and developing a robust regeneration protocol for antibody–antigen binding on the biosensor platform. Extending the platform to detect related analytes in complex matrices, developing portable devices for field deployment, and systematically investigating temperature effects on biosensor performance, including temperature cross-sensitivity, will also be priorities in future studies.

Although this study shows promising performance, some aspects of LSPR-based optical biosensing still need to be further investigated. The stability and reproducibility of plasmonic sensing interfaces are still important issues since variations in the functionalization of nanomaterials and the refractive index of the surrounding medium may affect the sensing response, especially in complex sample matrices. Moreover, long-term stability and regeneration of the functionalized sensing interfaces are important for repeated and practical applications. Future work will therefore be directed towards further optimization of the nanocomposite sensing interface, improvement of the long-term stability and regeneration performance, testing the biosensor in more complex real-world samples, and the integration of the tapered fiber-optic LSPR platform with compact and portable optical readout systems. These efforts can further improve the robustness, practicality and field-deployment potential of the proposed optical biosensing platform.

## Figures and Tables

**Table 1:** Comparison of the sensing performance of probes with different functional materials.

Biosensor Configuration	Sensitivity (nm/μM)	LoD (μM)
AuNPs	0.0132	20.2
AuNPs/MWCNTs	0.0290	9.20
AuNPs/WS_2_-QDs	0.0347	7.69
AuNPs/MWCNTs/WS_2_-QDs	0.0689	3.87

**Table 2:** Real samples detection results and recoveries of triclosan in real samples.

Samples	Added Triclosan (μM)	Recovery Rate (%)	RSD (%)
Toothpaste	20	87–109%	10.1%
	40	80–102%	11.3%
Facial cleanser	20	89–95%	9.4%
	40	91–114%	13.1%

**Table 3:** Performance comparison of the proposed biosensor with existing triclosan detection methods.

Mechanism	Materials Used	Linear Range	Sensitivity	LoD	Ref.
Electrochemistry	CuZn@NC	0.2–600 μM	n.r. ^a^	47.9 nM	[37]
Electrochemistry	Carbon black /SiO_2_-TiO_2_-Nb_2_O_5_	0.025–57.01 μM	n.r. ^a^	1.23 nM	[38]
Activated carbon composite	ZIF-11/Rice husk activated carbon	0.1–8 μM	n.r. ^a^	0.076 μM	[39]
Electrochemistry	Paper-based/graphite-silicone	0.20–10.30 μM	n.r. ^a^	0.05 μM	[40]
Electrochemistry	Eu_2_O_3_/MWCNT	0.38–18 μM	n.r. ^a^	2.3 nM	[41]
SERS	AgNP-decorated ZnO multipods	n.r. ^a^	n.r. ^a^	0.3 ppb	[42]
SERS	Au octahedra (AuOH)	2 ppb–5 ppm	n.r. ^a^	1.70 ppb (water); 1.91 ppb (mouthwash)	[43]
Ratiometric fluorescence + smartphone	CQDs@MIP	1.2–100 nM	n.r. ^a^	0.56 nM	[44]
LSPR	AuNPs/MWCNTs/WS_2_-QDs	0–40 μM	0.0689 μM	3.87 nm/µM	This work

^a^ not reported.

## Data Availability

Data underlying the results presented in this paper are not publicly available at this time but may be obtained from the authors upon reasonable request.
